# Prevalence and associated skills of Australian general practice registrars seeing children with functional bowel and bladder problems

**DOI:** 10.1111/jpc.16444

**Published:** 2023-05-26

**Authors:** Sharon Goldfeld, Amanda Tapley, Elodie O'Connor, Neil Spike, Simon Morgan, Gary L Freed, Andrew Davey, Elizabeth Holliday, Jean Ball, Parker Magin

**Affiliations:** ^1^ Centre for Community Child Health, Murdoch Children's Research Institute Royal Children's Hospital Melbourne Victoria Australia; ^2^ Department of Paediatrics University of Melbourne Melbourne Victoria Australia; ^3^ NSW and ACT Research and Evaluation Unit GP Synergy Newcastle New South Wales Australia; ^4^ School of Medicine and Public Health University of Newcastle Newcastle New South Wales Australia; ^5^ Eastern Victoria General Practice Training Melbourne Victoria Australia; ^6^ Department of General Practice University of Melbourne Melbourne Victoria Australia; ^7^ Child Health Evaluation and Research Center University of Michigan Ann Arbor Michigan USA; ^8^ Centre for Health Policy, Melbourne School of Population and Global Health University of Melbourne Melbourne Victoria Australia; ^9^ Clinical Research Design and Statistical Support Unit (CReDITSS) Hunter Medical Research Institute Newcastle New South Wales Australia

**Keywords:** child health, consultation, general practice education, health services

## Abstract

**Aim:**

Functional bowel (constipation and faecal incontinence) and bladder (urinary incontinence and enuresis) problems in children are often treated by paediatricians yet should mostly be managed by general practitioners (GPs). To understand whether the necessary skills and knowledge are being built in general practice, this study aimed to establish the prevalence and associated skills of Australian general practice registrars managing children with functional bowel and bladder problems. Together as paediatricians and GPs, we use these data to determine how best to ensure high quality, equitable care for children.

**Methods:**

We drew on 16 rounds of data collection from the Registrar Clinical Encounters in Training (ReCEnT) multi‐site cohort study (2010–2017) of general practice registrars' in‐consultation experience. It included a measure of paediatric consultations in which a functional bowel or bladder problem was managed, as well as demographic information.

**Results:**

Out of 62 721 problems/diagnoses for paediatric patients (0–17 years), 844 (1.4%) were coded as functional bowel (*n* = 709; 1.13% (95% confidence interval, CI: 1.05–1.22)) and/or bladder (*n* = 135; 0.22% (95% CI: 0.18–0.25)) presentations. Registrars were more likely to prescribe medication for bowel problems (odds ratio (OR) = 2.22 (95% CI: 1.86–2.64)) than for all other problems, but less likely to prescribe medication (OR = 0.31 (95% CI: 0.18–0.52)) for night‐time wetting and more likely to make a specialist referral (OR = 1.99 (95% CI: 1.22–3.25)) compared to all other problems.

**Conclusions:**

Only a small proportion of children with functional bowel and bladder problems were seen by registrars despite high prevalence in the community and amenability to management in the general practice setting (i.e. generally low morbidity and low complexity) versus need for specialists. Registrars appeared to be managing functional bowel and bladder problems according to evidence‐based guidelines, but with relatively high levels of referral. Given the inequitable access to specialist care, paediatricians should support local general practice management of these problems. This might include (i) engaging with training programs to ensure appropriate education and (ii) liaising with individual registrars/practices to provide management advice for individual or example cases.

## What is already known on this topic

1. The high prevalence and relatively low morbidity and complexity of paediatric functional bowel and bladder problems suggest that general practitioners are well‐placed to address these issues and that they do not require specialist paediatric care.
2. General practice registrars see a limited number and range of paediatric patients within their apprenticeship‐like model of vocational training. The implication is that the burden of management for these mostly straightforward conditions often falls upon specialist paediatric services.

## What this paper adds

1. The rate of contact for general practice registrars seeing functional bowel and bladder problems within paediatric encounters was well below what might be expected given high community prevalence.
2. There was some evidence of registrar actions aligning with evidence‐based guidelines when functional bowel and bladder problems were encountered. However, the rate of referral to specialty care appeared to be relatively high.
3. Despite the low exposure of general practice registrars to children with functional bowel and bladder problems, the high rates of specialist referral should encourage paediatricians to support general practice management of these problems.

In Australia, general practitioners (GPs) are the most commonly accessed health practitioners by children.^1^ GPs provide relatively equal access for children across socio‐economic groups; while this does not equate with equitable care – given higher rates of morbidity in children of lower socio‐economic status^2^ – access to GPs is still far more equitable than access to specialist paediatric care, where there is a significant social gradient.^3^ GPs therefore play an increasingly important role in providing equitable care to children, especially for conditions that can be managed in primary care settings. Given that GPs are the gatekeepers to specialist care, there is some concern regarding the increasing rate of referral to paediatric secondary and tertiary care.^4^ There undoubtedly may be systemic issues in equitable access to specialist paediatric care; however, the imperative is for GPs to manage GP‐appropriate conditions (rather than referring them). The increasing rate of referral may be due to difficulty keeping up to date with the myriad of conditions needed to be managed,^5^ or that the time required to manage complex chronic conditions may be a disincentive to provide such care.^6^ Nevertheless, it should be in the interest of paediatricians to have children's health needs met by the most equitable health‐care system available.^7^

In order for GPs to manage paediatric patients, they must be well‐trained and well‐skilled, placing a burden on GP registrars and those responsible for their training. As the duration of registrar training has not increased in recent decades and structural factors increasingly limit junior hospital doctor acquisition of GP‐relevant knowledge and skills,^8^ more information and potentially more educational experiences must be included within existing time constraints.^9^ Perhaps exacerbating the issue is whether registrars at the beginning of their GP career are gaining sufficient clinical exposure, and therefore confidence, in caring for children during the apprenticeship‐like model of training.^4^

Functional bowel and bladder problems are a good example of conditions that should be amenable to management in primary care. Bowel and bladder problems in children include a range of conditions which together are relatively prevalent. These include common functional conditions such as constipation, with prevalence rates ranging from 1 to 30%^10^; urinary incontinence (daytime wetting) with a 5% prevalence rate, and enuresis (bed wetting), affecting up to 19% of Australian children^11^ (see Table 1 for definitions). They also include rarer structural conditions (e.g. Hirschsprung's disease), which require specialist care. Bowel and bladder problems reduce the quality of life for both the parent and child involved,^12^ and can lead to behavioural problems, anxiety and low self‐esteem.^13^ These problems are also commonly associated with more significant medical problems, such as vesicoureteral reflux and urinary tract infections, which can lead to more complicated health issues.^14^

**Table 1:** Definitions of functional bowel and bladder problems

	Definition
**Functional bowel problems** †	
Constipation	Must include 2 or more of the following: ≤2 defecations per week; ≥1 episode of faecal incontinence per week; history of retentive posturing or excessive volitional stool retention; history of painful or hard bowel movements; presence of large faecal mass in rectum; history of large diameter stools which may obstruct toilet
Faecal incontinence (bowel incontinence and encopresis)	Must include all of the following: defecation into places inappropriate to social context (including underwear) at least once per month; no evidence of inflammatory, anatomic, metabolic, or neoplastic process that explains symptoms; no evidence of faecal retention
Faecal impaction	Severe constipation with a large faecal mass in either the rectum or the abdomen, and/or overflow soiling
**Functional bladder problems** ‡	
Daytime problems (urinary incontinence and diurnal enuresis)	Involuntary leakage of urine Symptom requires a minimum age of 5 years, a minimum of one episode per month and a minimum duration of 3 months to be termed a condition Is a significant condition if it occurs >1 episode per month and a frequency of 3 episodes over 3 months
Night‐time problems (bedwetting, enuresis and nocturnal enuresis)	Is both a symptom and a condition of intermittent incontinence that occurs during periods of sleep Is a significant condition if it occurs >1 episode per month and a frequency of 3 episodes over 3 months

† Source: Rome Foundation Diagnostic Algorithms. Appendix B: Rome III Diagnostic Criteria for Functional Gastrointestinal Disorders. *Am. J. Gastroenterol*. 2010;105:798–801.

‡ Source: Austin PF, Bauer SB, Bower W, Chase J, Franco I, Hoebeke P, *et al*. The standardisation of terminology of lower urinary tract function in children and adolescents: update report from the Standardisation Committee of the International Children's Continence Society. *J Urol*. 2014;191(6):1863–5.e13.

Of all these problems, the high prevalence and relatively low morbidity and low complexity of functional (as opposed to structural) bowel and bladder problems means GPs are both well‐placed and should be well trained and competent to care for these children.^15^ Supporting this suggestion are several evidence‐based guidelines for the management of functional bowel and bladder problems. These have been recently updated for Australia and are well‐suited and specifically written for care outside a secondary or tertiary setting.^16^ For example, for bladder problems such as enuresis, it is recommended that non‐pharmacological interventions (e.g. a bell and pad alarm) are attempted before pharmacological therapies.^17^ For bowel problems such as constipation, it is recommended that behavioural and dietary modifications are utilised alongside laxatives.^16^

Despite the amenability of these problems to primary care, there appears to be a dissonance of practice, with evidence showing Victorian hospital admission rates for constipation are 10‐fold higher than in the USA.^18^ This may be due to the USA having different health systems and admission policies,^18^ including specialist paediatricians sited in primary care, which may prevent some patients needing hospital‐located specialist paediatric care. If the care that children can receive from primary care paediatricians in the USA is difference, then this supports our argument that Australian GPs skills in this area should be enhanced. Further, children referred to public outpatient clinics are often on wait lists for several months.^19^, ^20^ The implication is that the burden of care for these mostly straightforward but chronic paediatric conditions falls upon specialist paediatric services. And the resultant question is, ‘how efficiently is the GP gatekeeper‐to‐specialist‐care function operating?’ There are, however, few data available to understand the true nature of GPs' approach to functional bowel and bladder problems – the point of entry for these patients into overstretched and inequitable specialist services.

To address this gap, and potentially direct more management of these conditions into general practice, we need to better understand GP practice regarding functional bowel and bladder problems. Therefore, we aimed to establish the prevalence of functional bowel and bladder problems in children and the associated skills of Australian GP registrars. We also specifically sought to establish the relative frequency and the nature of consultations for specialist referral and medication prescriptions for functional bowel and bladder problems.

## Methods

### Data source

Sixteen rounds of 6‐monthly data collection (2010–2017) of the Registrar Clinical Encounters in Training (ReCEnT) ongoing multi‐site cohort study of the within‐consultation experiences of GP registrars. Study participants included registrars training across five states and the ACT, with five Regional Training Providers (RTPs; 2010–2015) and three Regional Training Organisations (RTOs; 2016–2017; there was a major restructure of Australian GP training in 2015/2016).

The ReCEnT study methodology has been published elsewhere.^21^ Briefly, registrars undertake data collection on three occasions, 6‐monthly, during their mandatory community training terms. Initial data collection for each registrar, each training term, involved questionnaire items relating to their demographics, education and work experience. Registrars then recorded the details of 60 consecutive clinical consultations per term on a paper‐based encounter form. As data collection was designed to reflect a ‘normal’ week of general practice, consultations in a specialised clinic (e.g. vaccination clinic) were excluded. Only office‐based consultations (not home visits or nursing home visits) were recorded.

### Measures

#### Primary aim/outcomes

The primary outcome was whether a diagnosis or problem (hereafter, ‘problem’) was a functional bowel or bladder condition (structural conditions were excluded). Problems were coded according to the International Classification of Primary Care, second edition (see Table 1 for further detail).

#### Main explanatory variables of interest

Referral and medication prescription were included as the main explanatory variables of interest in the analyses.

#### Additional explanatory variables

*Registrar‐specific variables* included age, gender, whether they graduated in Australia, and their RTP/RTO‐defined region/subregion (hereafter, ‘region’).

*Registrar‐term and practice variables* included registrar's training term (1, 2 or 3), whether the registrar had worked at the practice previously, full‐time or part‐time status, practice billing policy (whether the practice routinely bulk‐bills all patients), and practice size (number of full‐time equivalent GPs). Practice postcode was used to define practice (i) rurality as per the Australian Standard Geographical Classification‐Remoteness Area classification and (ii) Socio‐Economic Indexes for Areas (SEIFA) Relative Index of Disadvantage decile.

*Patient variables* included age, gender, Aboriginal or Torres Strait Islander status, non‐English speaking background and the patient being new to the practice or to the registrar.

*Consultation variables* related to (i) consultation content: whether it was a new problem, duration and number of problems addressed, and whether the registrar sought in‐consultation advice or information from their supervisors or other resources, such as specialists, books or electronic resources; and (ii) consultation outcome: whether pathology or imaging tests were ordered, referral or follow‐up organised, medication prescribed, or learning goals generated for post‐consultation attention.

### Statistical methods

The proportion of paediatric (age 0–17 years) problems that were functional bowel and bladder problems were calculated with 95% confidence intervals (CIs), using standard error estimates adjusted for repeated measures within registrars. Analyses were performed to test the univariate and adjusted associations of a paediatric consultation being a functional bowel and/or bladder problem. Logistic regression was used within the generalised estimating equations framework to account for repeated measures within registrars. Univariate analyses were conducted on each covariate, with the outcome. Covariates with a univariate *P* value < 0.2 were considered for inclusion in the multiple regression models. Once the model with all significant covariates was fitted, model reduction was assessed. Covariates which were no longer significant (at *P* < 0.2) in the multi‐variable model were tested for removal from the model. If the covariate's removal did not substantively change the resulting model, the covariate was removed from the final model. All analyses were undertaken using Stata 14.0 and SAS 9.4. The project has approval from the relevant Human Research Ethics Committee.

## Results

A total of 1737 registrars (response rate 96.2%) from 728 practices contributed 4074 trainee‐rounds of data, including details of 242 008 patient encounters (and 378 189 problems). Demographic characteristics are presented in Table 2. Of all consultations recorded, 49 250 (20.4% (95% CI: 20.2–20.5)) were for paediatric patients (0–17 years); with 62 721 problems recorded.

**Table 2:** Participating registrar, registrar‐term and practice‐term demographics

Variable	Class	*n* (%)
**Registrar variables (*n* = 1737)**		
Gender	Female	1114 (64.1)
Graduated in Australia	Yes	1423 (82.5)
**Registrar‐term and practice‐term variables (*n* = 4074**)		
Registrar age (years)	Mean (SD)	32.4 (6.1)
Registrar training term	Term 1	1615 (39.6)
	Term 2	1470 (36.1)
	Term 3	989 (24.3)
Registrar worked at practice previously	Yes	994 (24.7)
Registrar works full‐time	Yes	3079 (77.7)
Fully bulk billing practice	Yes	895 (22.3)
Number of GPs working at practice	5 or more	2527 (63.9)
Rurality of practice	Major city	2444 (60.2)
	Inner regional	1024 (25.2)
	Outer regional or remote	595 (14.6)
SEIFA† index	Mean (SD)	5.6 (2.9)

† Socio‐Economic Indexes For Areas (SEIFA) Relative Index of Disadvantage. Higher SEIFA scores indicated less disadvantage.

GP, general practitioner; SD, standard deviation.

Of all paediatric problems, 844 (1.3% (95% CI: 1.3–1.4)) were coded as a functional bowel (*n* = 709; 1.13% (95% CI: 1.05–1.22)) or bladder (*n* = 135; 0.22% (95% CI: 0.18–0.25)) problem (Table 3). The overlap of bowel and bladder problems addressed at the same consultation was small (*n* = 8; 1.1% of bowel problems and 5.9% of bladder problems). A total of 606 individual registrars saw these 844 functional bowel or bladder problems. Registrars saw between one (72%) and five of these problems within their encounters (20% saw two; 5% saw three and 2% saw four or five).

**Table 3:** Types of functional bowel and bladder problems

	*n* (%)
**Functional bowel problems (*N* = 709)**	
Constipation	665 (93.8)
Faecal incontinence	44 (6.2)
**Functional bladder problems (*N* = 135)**	
Daytime problems	38 (28.2)
Night‐time problems	97 (71.8)

The percentage of functional bowel and functional bladder problems prescribed medication was 63% and 17%, respectively (Table 4). The most commonly prescribed medications were Macrogol (including combinations) for functional bowel problems and Desmopressin and Oxybutynin for functional bladder problems. The percentage of functional bowel and functional bladder problems referred to specialty care was 11% and 30%, respectively. The most common referrals made were to paediatricians for both functional bowel and bladder problems.

**Table 4:** Distributions of patient, registrar, practice and consultation variables according to the presence/absence of a functional bowel or bladder problem in paediatric patients

		Functional bowel problems			Functional bladder problems		
		No (*n* = 62 012)	Yes (*n* = 709)		No (*n* = 62 586)	Yes (*n* = 135)	
Variable	Class	*n* (%)	*n* (%)	*P*	*n* (%)	*n* (%)	*P*
**Medications and referrals**							
Medication prescribed	Yes	28 157 (45.4)	447 (63.1)	<0.0001	28 581 (45.7)	23 (17.0)	<0.0001
Referral ordered	Yes	6405 (10.3)	75 (10.6)	0.85	6439 (10.3)	41 (30.4)	<0.0001
**Additional variables**							
*Patient variables*							
Patient age group	0–3 years	23 995 (38.7)	313 (44.2)	<0.0001	24 288 (38.8)	20 (14.8)	<0.0001
	4–10 years	18 698 (30.2)	283 (39.9)		18 885 (30.2)	96 (71.1)	
	11–17 years	19 319 (31.2)	113 (15.9)		19 413 (31.0)	19 (14.1)	
Patient gender	Female	31 846 (52.3)	375 (54.0)	0.36	32 148 (52.3)	73 (55.3)	0.53
Aboriginal or Torres Strait Islander	Yes	1386 (2.4)	12 (1.8)	0.36	1395 (2.4)	3 (2.3)	1.00
Non‐English speaking background	Yes	3924 (6.7)	51 (7.6)	0.46	3969 (6.7)	6 (4.7)	0.40
Patient/practice status	Existing patient	19 276 (31.7)	252 (36.3)	0.03	19 488 (31.8)	40 (29.9)	0.07
	New to registrar	36 033 (59.3)	380 (54.8)		36 324 (59.3)	89 (66.4)	
	New to practice	5431 (8.9)	62 (8.9)		5488 (9.0)	5 (3.7)	
*Registrar variables*							
Registrar age	Mean (SD)	32.2 (6.1)	31.8 (5.5)	0.09	32.2 (6.1)	31.6 (5.7)	0.23
Registrar gender	Female	39 750 (64.1)	465 (65.6)	0.45	40 120 (64.1)	95 (70.4)	0.15
Registrar works full‐time	Yes	46 936 (77.5)	526 (75.9)	0.42	47 363 (77.5)	99 (74.4)	0.38
Term	Term 1	24 694 (39.8)	271 (38.2)	0.63	24 909 (39.8)	56 (41.5)	0.92
	Term 2	22 485 (36.3)	268 (37.8)		22 706 (36.3)	47 (34.8)	
	Term 3	14 833 (23.9)	170 (24.0)		14 971 (23.9)	32 (23.7)	
Worked at practice previously	Yes	14 458 (23.6)	165 (23.5)	1.00	14 596 (23.6)	27 (20.2)	0.38
Graduated in Australia	Yes	51 512 (83.6)	622 (88.1)	0.002	52 009 (83.6)	125 (92.6)	0.005
Region	1	15 457 (24.9)	165 (23.3)	0.42	15 586 (24.9)	36 (26.7)	0.97
	2	5391 (8.7)	68 (9.6)		5449 (8.7)	10 (7.4)	
	3	8092 (13.1)	83 (11.7)		8160 (13.0)	15 (11.1)	
	4	26 578 (42.7)	327 (46.1)		26 847 (42.9)	58 (43.0)	
	5	1727 (2.8)	15 (2.1)		1738 (2.8)	4 (3.0)	
	6	4767 (7.7)	51 (7.2)		4806 (7.7)	12 (9.0)	
*Practice variables*							
Practice size	Large	40 332 (66.8)	476 (68.7)	0.31	40 721 (66.8)	87 (65.9)	0.80
Rurality	Major city	38 655 (62.5)	470 (66.5)	0.12	39 035 (62.5)	90 (67.2)	0.36
	Inner regional	14 898 (24.1)	155 (21.9)		15 021 (24.1)	32 (23.9)	
	Outer regional or remote	8309 (13.4)	82 (11.6)		8379 (13.4)	12 (9.0)	
SEIFA† index	Mean (SD)	5.9 (2.9)	5.9 (3.0)	0.59	5.9 (2.9)	6.5 (2.8)	0.011
Practice routinely bulk bills	Yes	12 545 (20.5)	141 (20.4)	0.99	12 664 (20.6)	22 (17.1)	0.32
*Consultation variables*							
Consultation duration	Mean (SD)	16.8 (8.6)	19.8 (8.7)	<0.0001	16.8 (8.6)	20.0 (7.9)	<0.0001
Number of problems	Mean (SD)	1.5 (0.8)	1.7 (0.8)	<0.0001	1.5 (0.7)	1.6 (0.8)	0.10
Is a new problem	Yes	41 450 (73.8)	377 (59.3)	<0.0001	41 775 (73.7)	52 (41.3)	<0.0001
Sought help any source	Yes	12 687 (20.5)	201 (28.4)	<0.0001	12 838 (20.5)	50 (37.0)	<0.0001
Pathology ordered	Yes	5993 (9.3)	45 (5.6)	0.006	5979 (9.2)	59 (43.0)	<0.0001
Imaging ordered	Yes	2885 (4.7)	32 (4.5)	0.85	2901 (4.6)	16 (11.9)	0.0002
Follow‐up ordered	Yes	24 390 (39.3)	362 (51.1)	<0.0001	24 682 (39.4)	70 (51.9)	0.004
Learning goals generated	Yes	10 568 (17.9)	179 (26.6)	<0.0001	10 680 (17.9)	67 (52.8)	<0.0001

† Socio‐Economic Indexes For Areas (SEIFA).

SD, standard deviation.

On multi‐variable analyses (Table 5), registrars were significantly more likely to prescribe medication for functional bowel problems than for all other problems (OR = 2.22; (95% CI: 1.86–2.64)), but there was no significant association with specialist referral compared to other problems. For functional bladder problems, registrars were significantly less likely to prescribe medication (OR = 0.31; (95% CI: 0.18–0.52)), and more likely to make a specialist referral (OR = 1.99; (95% CI: 1.22–3.25)).

**Table 5:** Adjusted odds ratios (ORs) representing associations between independent variables and functional bowel or bladder problems

		Functional bowel problems		Functional bladder problems	
Variable	Class	Adjusted OR (95% CI)	*P*	Adjusted OR (95% CI)	*P*
**Medications and referrals**					
Medication prescribed	No	Ref		Ref	
	Yes	2.22 (1.86–2.64)	<0.0001	0.31 (0.18–0.52)	<0.0001
Referral ordered	No	–		Ref	
	Yes	–		1.99 (1.22–3.25)	0.006
**Additional variables**					
*Patient variables*					
Patient age group	0–3	Ref		Ref	
	4–10	1.15 (0.96–1.39)	0.13	4.56 (2.58–8.07)	<0.0001
	11–17	0.35 (0.27–0.45)	<0.0001	0.64 (0.30–1.36)	0.25
Patient/practice status	Existing patient	–		Ref	
	New to practice	–		0.80 (0.32–2.02)	0.63
	New to registrar	–		1.70 (1.10–2.62)	0.017
*Registrar variables*					
Graduated in Australia	No	Ref		Ref	
	Yes	1.56 (1.18–2.07)	0.002	2.07 (1.06–4.07)	0.034
*Practice variables*					
SEIFA† index		–		1.08 (1.00–1.15)	0.045
*Consultation variables*					
Consultation duration		1.03 (1.02–1.03)	<0.0001	1.01 (0.99–1.02)	0.32
Number of problems		1.31 (1.19–1.45)	<0.0001	–	
Is a new problem	No	Ref		Ref	
	Yes	0.48 (0.40–0.58)	<0.0001	0.29 (0.19–0.44)	<0.0001
Imaging ordered	No	–		Ref	
	Yes	–		2.21 (1.22–3.99)	0.009
Follow‐up ordered	No	Ref		–	
	Yes	1.51 (1.27–1.81)	<0.0001	–	
Learning goals generated	No	Ref		Ref	
	Yes	1.55 (1.26–1.90)	<0.0001	3.12 (2.07–4.70)	<0.0001
Pathology ordered	No	Ref		Ref	
	Yes	0.60 (0.42–0.84)	0.003	6.84 (4.49–10.4)	<0.0001

† Socio‐Economic Indexes For Areas (SEIFA).

CI, confidence interval.

## Discussion

In this study, we were able to determine that Australian GP registrars experienced a relatively low rate of functional bowel and bladder problems encounters despite the high community prevalence. In a typical 18‐month training program, most registrars would see few children with functional bowel problems (on average 14) and minimal children with functional bladder problems (on average 3). This may have a significant impact on registrars' confidence to provide care to these children. Previous research has shown that Australian GP registrars have far less confidence in managing non‐acute conditions (including ‘soiling/wetting and constipation’) than acute conditions.^22^

Nevertheless, there was some evidence of registrars managing functional bowel and bladder problems according to clinical practice guidelines.^16^, ^17^ This is despite, or perhaps because, registrars find these areas challenging (being more likely to generate learning goals around managing functional bowel and bladder problems compared with other paediatric problems). Clinical practice guidelines for functional bowel problems recommend the use of medications such as oral laxatives for constipation^16^; there was relatively high use of prescribing laxatives suggesting appropriate management by registrars. Guidelines for functional bladder problems recommend utilising bedwetting alarms (night‐time), and reward systems and toileting routines with potential referral as required (daytime); prescribed medications are generally not recommended.^17^ With the caveat of small numbers of encounters, the relatively low use of prescribed medication (compared to other paediatric problems) may also suggest appropriate management by registrars. The higher rates of registrars organising follow‐up is encouraging given that these problems often require longer term management.^16^, ^17^

However, the rates of referral appear to be relatively high (11% of functional bowel problems and 30% of functional bladder problems referred to specialty care). In addition, the rates of imaging being ordered were also relatively higher than expected (4.5% of functional bowel problems and 11.9% of functional bladder problems), particularly for functional bladder problems. Given these rates should be almost zero in GP practice, this indicates the need to upskill GP registrars (and potentially, associated GP practices), to better implement best practice guidelines.

It is important not to over‐medicalise issues, but these functional conditions cause considerable distress and are highly amenable to treatment. The key issue is – of those who present, a significant proportion get referred. The paediatricians who see these referrals are very confident the great majority can be managed by a skilled GP. When registrars lack confidence,^22^ this can result in high rates of referral, which then puts strain on secondary care services with long wait times for children with these easily treated conditions. This bottleneck likely also leads to delays in the management of other children on the wait list with conditions more needing specialist management. The long wait lists cannot be attributed only to registrar inexperience: they reflect wider GP referral patterns and it is reasonable to assume that registrars' referral patterns to a large degree reflect the patterns of the practice in which they are training. The backlog of paediatric referral cases is not only a workload issue for paediatricians, but an unnecessary delay in symptom management for distressed children and their parents.

However, there remains a dissonance between our results with modest referral rates, and the significant burden of conditions such as constipation in hospital outpatient and emergency departments.^1^, ^23^ Also puzzling is the apparent relatively low prevalence of these conditions in registrars' practice. Previous work has shown that this study represents a very similar sample of problems that registrars are seeing to that of established GPs.^24^ Structural practice‐level approaches to patient scheduling could preferentially direct non‐acute paediatric conditions to registrars and help registrars (with supervisor oversight) develop the needed management skills.^1^

Together as paediatricians and GPs, our aim was to use these data to determine how best to ensure high quality, equitable care for children. Given the inequitable and often difficult access to specialist paediatric care, it makes sense that paediatricians are at the forefront of ensuring that GPs have the right skills and support to be excellent clinicians and gatekeepers for these children. Proactive paediatrician initiatives at the local level could provide defined and accessible routes to advice for GPs regarding functional bowel and bladder problems. This might include engaging with GP vocational training programs (especially locally) to ensure that GP registrars (and their supervisors, given the apprenticeship‐like training model) are provided with appropriate education on this common developmental topic. Building these relationships would mean that individual GP registrars or practices could then liaise with individual local paediatricians regarding management advice for individual or example cases, rather than resorting immediately to formal referral. This approach has been tested in the UK^25^ and is now being trialled in Australia for a range of high‐prevalence conditions (particularly behavioural/developmental) that can be managed by GPs and prevent long wait lists for families.^26^

This study has several strengths. It collected data from a large number of registrars across a broad range of locations. The response rate was high for a study of GPs^27^ and inclusion of data from five Australian states across all rurality classifications, from major cities to very remote areas, suggests generalisability to the wider Australian GP vocational training program. The primary limitation of the study was the cross‐sectional nature of the data which precludes inferences of causality in the associations found. We also did not have data on medication regimens or previous referrals. The relatively small number of functional bladder problems limited our ability to derive firm conclusions about their associations; especially differentiating between daytime and night‐time wetting. A further consideration is that our main explanatory variables of interest were medication prescription and referral. Significant associations of independent variables in our multi‐variable analysis are of interest and may aid interpretation of our findings regarding medications and referrals but need confirmation in further studies.

Chronic conditions such as functional bowel and bladder problems impact on children's use of health services. This includes more expensive services such as hospital outpatient and emergency departments.^1^, ^23^ For example, over a 3‐month period, 18.9% of children who presented to Victorian hospital outpatient clinics had been referred for a primary diagnosis of functional bowel and bladder problems.^20^ The potential economic burden associated with hospitalisation^23^ (e.g. constipation has been estimated to cost Victorian public hospitals $5.5 million per year) reinforces the need for both better public education and education of parents more generally.

Socio‐economic causes likely impact referrals to public hospital outpatient clinics and will be exacerbated by funding and renumeration constraints on GPs' ability to deliver best‐practice care to patients of lower socio‐economic status. However, access to comprehensive primary care is the best means of attenuating the effects of socio‐economic status on health,^2^ given the inequitable access to specialist paediatric care.^3^ Despite the current funding constraints on general practice, this highlights the potential for effective and appropriate GP care, with the resultant reduction of system costs for avoidable hospitalisations and emergency department visits.^1^ Families' access to paediatric care^28^ is limited by potential out‐of‐pocket costs plus long wait times for paediatricians (especially as a result of delayed care during the pandemic), even in public outpatient clinics. Therefore, the need to ensure children can be seen and managed by local GPs for common chronic conditions such as functional bowel and bladder problems is more important than ever.

## Conclusions

Functional bowel and bladder problems in children were seen relatively infrequently by GP registrars despite high prevalence in the community, their chronic nature and amenability to GP management. The encouraging level of evidence‐based management of these problems by registrars, despite the higher referral rates, should encourage paediatricians to support local general practice management of functional bowel and bladder problems.

## Ethics statement

The project has approval from the University of Newcastle Human Research Ethics Committee, Reference H‐2009‐0323.

## Data Availability

The data underlying this article cannot be shared publicly due to ethics requirements which protect the privacy of individuals that participated in the study.
